# Comparative Analysis of Whole-Genome Gene Expression Changes in Cultured Human Embryonic Stem Cells in Response to Low, Clinical Diagnostic Relevant, and High Doses of Ionizing Radiation Exposure

**DOI:** 10.3390/ijms160714737

**Published:** 2015-06-30

**Authors:** Mykyta Sokolov, Van Nguyen, Ronald Neumann

**Affiliations:** Nuclear Medicine Division, Department of Radiology and Imaging Sciences, Clinical Center, National Institutes of Health, Bethesda, MD 20892, USA; E-Mails: van.nguyen3@nih.gov (V.N.); rneumann@mail.nih.gov (R.N.)

**Keywords:** whole genome, human embryonic stem cell, ionizing radiation, DNA microarray, low dose exposure

## Abstract

The biological effects of low-dose ionizing radiation (LDIR) exposure in humans are not comprehensively understood, generating a high degree of controversy in published literature. The earliest stages of human development are known to be among the most sensitive to stress exposures, especially genotoxic stresses. However, the risks stemming from exposure to LDIR, particularly within the clinical diagnostic relevant dose range, have not been directly evaluated in human embryonic stem cells (hESCs). Here, we describe the dynamics of the whole genome transcriptional responses of different hESC lines to both LDIR and, as a reference, high-dose IR (HDIR). We found that even doses as low as 0.05 Gy could trigger statistically significant transient changes in a rather limited subset of genes in all hESCs lines examined. Gene expression signatures of hESCs exposed to IR appear to be highly dose-, time-, and cell line-dependent. We identified 50 genes constituting consensus gene expression signature as an early response to HDIR across all lines of hESC examined. We observed substantial differences in biological pathways affected by either LDIR or HDIR in hESCs, suggesting that the molecular mechanisms underpinning the responses of hESC may fundamentally differ depending on radiation doses.

## 1. Introduction

Despite decades of dedicated research, the studies into the biological effects of low doses (LD) of ionizing radiation (IR) continue to produce contradictory results; no broad consensus has been reached regarding health effects and risk assessment in humans for doses in a range below 0.1 Gy [1,2,3,4,5,6]. The background radiation sources from cosmic rays, Earth’ crust radioisotopes, and radiation sources stemming from various types of human activities, such as radioactive waste, radiologic accidents, clinical diagnostic computerized tomography (CT) scans all contribute to LDIR human exposures. LDIR exposures are essentially unavoidable, which is in marked contrast to much rarer incidences of high doses (HD) of IR exposures in humans arising mostly from IR therapeutic cancer treatments. Published data imply that doses of IR more than 0.2 Gy may increase the risks for cancer and other pathologies [7]. However, it was reported that the use of CT scans in children to deliver LDIR of about 0.05 Gy might almost triple the risk of leukemia and doses of about 0.06 Gy might triple the risk of brain cancer [8]. The numbers of CT scans have increased rapidly worldwide, especially in the past 10 years, raising health concerns [9]. The uncertainties of predicting the biological effects of LDIR exacerbate the known phenomena of non-targeted effects of IR; among them are radioadaptive responses, radiation-induced bystander effects and LDIR hypersensitivity. Some researchers posit that there are no risks, or even potential beneficial hormetic effects, associated with at least some LDIR exposures [1].

The gene expression changes were firmly established as an early indicator of cellular responses to LDIR in humans [10,11]. Genome-wide DNA microarray analysis was applied to study the effects of LDIR, and low dose rate IR in a number of experimental models thus far [12,13,14,15,16,17,18]. These reports highlighted a high degree of variability in LDIR responses in humans across different genotypes, cell types, tissue types, and other experimental conditions. In a present study, to overcome the issue of differential IR-induced global gene expression changes among various normal human cells, we applied a novel human embryonic stem cell (hESC)-based culture model to examine LDIR in human cells. The data on how hESCs respond to IR at the transcriptome level are still scarce [19,20,21]. Moreover, no published data exist on LDIR global gene expression alterations of hESC.

We performed a study to directly assess the question of whether LDIR exposures within the clinical diagnostic relevant dose range result in genomic changes in hESCs at the level of whole transcriptome. Here, our goal was to comprehensively examine whole genome gene expression changes in a panel of several hESC lines following exposures to LDIR dose of 0.05 Gy. As a positive control for reference, we also used the HDIR of 1 Gy. We analyzed the dynamics of transcriptomic alterations at 2 and 16 h post-IR exposures, representing “early” and “late” hESC radioresponses, correspondingly. Our findings suggest both a temporal- and a hESC line-dependence of genomic radioresponses within the range of LDIR by these hESCs. In addition, the biological processes/pathways/themes implicated in a radioresponse of hESCs and affected specifically by LDIR were identified; these novel findings deepen our understanding of molecular mechanisms underpinning the biological effects of LDIR highly relevant to radiation exposures of very large cohorts of people worldwide.

## 2. Results and Discussion

**Figure 1:**
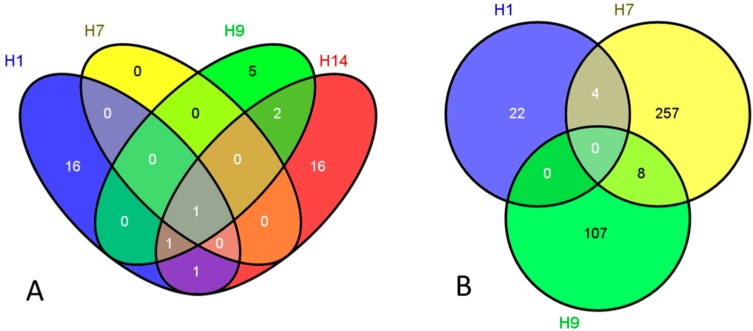
Venn diagrams of differentially expressed genes following low-dose ionizing radiation (LDIR) exposures: (**A**) upregulated genes and (**B**) repressed genes.

In our present study we set out (i) to compare the whole-genome gene expression changes in cultured hESCs in response to either LDIR, clinical diagnostic relevant, or HDIR of IR exposures; (ii) to examine “early” and “late” response of hESC to IR exposures; and (iii) to identify differences and similarities in response to both LDIR and HDIR across different hESC lines. Whole-genome microarray analysis using samples of total RNA extracted from cultures of H1, H7, H9 and H14 lines of hESCs indicated that only one gene (*CDKN1A*) is statistically significantly upregulated after 2 h post-LDIR exposures (0.05 Gy dose) in all four hESC lines examined (Figure 1A). *CDKN1A*, probably one of the best-studied IR-responsive genes [22,23], encodes p21, involved predominantly in cell cycle arrest through inhibition of cyclin dependent kinases [24]. Published data concerning the expression of *CDKN1A* in IR-exposed hESCs are, to a large extent, contradictory. As an example, Stein’s group reported that *CDKN1A* is upregulated either about 250-fold [25] or only 15-fold [26] in H1 line of hESCs 2 h post-5 Gy of IR compared to unirradiated controls. It was shown in some other studies that *CDKN1A* overexpression is observed only after HDIR (2–4 Gy, about 2–2.3-fold compared to mock-exposed references); and the relatively modest doses of IR (0.4 Gy) fail to elicit the upregulation of *CDKN1A* in H9 line of hESCs [19]. However, our published data suggested that a dose of 1 Gy of HDIR is enough to trigger a major induction of *CDKN1A* in H9 line of hESCs (almost 6-fold at 2 h post-IR, and 1.9-fold at 16 h post-1Gy) [20]. Our present results imply that the upregulation of *CDKN1A* gene expression is clearly detectable in all four hESCs examined even after LDIR as low as 0.05 Gy at 2 h post-exposure; however, the magnitude of response varied from 1.5-fold in H1 and H7 lines of hESCs to 2.1-fold over non-IR exposed cells in H9 line.

The vast majority of genes upregulated as an “early” radioresponse of hESCs to LDIR (0.05 Gy; 2 h) appears to be cell line-specific. For example, 16 genes out of 19 genes are unique to H1 hESC line (Figure 1A); and 16 genes out of 21 genes appear to be upregulated only in H14 hESC line compared to other four lines studied. Importantly, almost all LDIR-downregulated genes in hESCs studied are found to be cell line-specific too (Figure 1B). For example, out of 26 genes repressed following LDIR in H1 line of hESC, 22 genes are unique; out of 269 genes downregulated in H7 line of hESC, 257 genes are distinct; and out of 115 genes repressed in H9 hESCs 108 genes are found to be differentially expressed exclusively in this particular line. Interestingly, we found no downregulated genes in H14 line of hESCs after LDIR.

Our previous data showed that the IR-responsive gene expression changes in hESCs triggered by LDIR are modest at best [11]; in the present study, we confirmed and extended this finding utilizing the whole genome approach. We determined that the early gene expression changes in hESCs elicited a minor upregulation of a rather limited subset of genes, the vast majority of which were previously identified as being IR-responsive. Among these genes are *GDF15*, *BTG2* (induced in all lines of LDIR-exposed hESCs examined, except H7); *PLK2*, *SESN1* (upregulated in H9 and H14 lines of hESCs) [27,28,29,30]. Importantly, we observed lack of genes differentially expressed in hESCs following LDIR as a late response (0.05 Gy; 16 h) except H7 line of hESCs. This underscores that LDIR-induced transcriptional changes might be not only minor in scale, but also temporary. Even in H7 line of hESCs the two sequences identified at 16 h post-LDIR belong to poorly characterized transcripts, with no ascribed biological role whatsoever. Therefore, the whole genome DNA microarray approach is capable of detecting statistically significant changes in gene expression in hESCs even after doses of IR exposures as low as five rad (LDIR), almost exclusively as an integral part of an early radioresponse in these cells.

**Figure 2:**
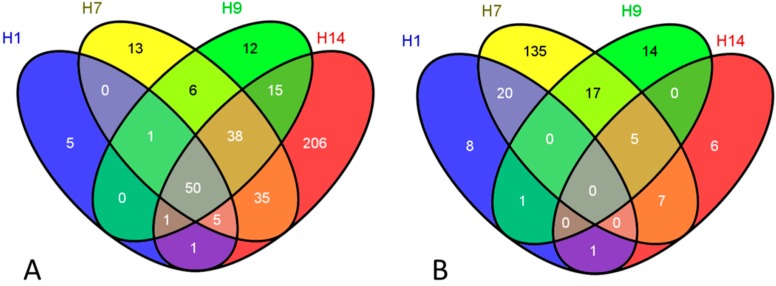
Venn diagrams of differentially expressed genes following high-dose ionizing radiation (HDIR) exposures (2 h): (**A**) induced genes and (**B**) repressed genes.

Our rigorous statistical analysis with DNA microarrays revealed that in total 63 genes were up-regulated in H1 hESCs, 148 genes induced in H7 hESCs, 123 genes in H9 hESC, and 351 genes overexpressed in H14 line of hESCs as an “early” response (2 h) following HDIR exposures (Figure 2A). We found 50 genes shared in common by all four hESCs examined under these experimental conditions; among these genes are well-established IR-responsive genes such as *GDF15*, *BTG2*, *PLK2*, *CDKN1A*, *PHLDA3*, *SESN1*, *BBC3*, *GADD45A*, *etc*. [27,28,29,30]. Some of these genes were also induced by much lower dose of IR (0.05 Gy), however, the magnitude of induction following HDIR was much more dramatic. For example, after HDIR exposures the level of overexpression of *GDF15* is 23.8-fold over sham-irradiation, whereas the level of induction of this gene following LDIR is only 2.1-fold in H9 line of hESC. *GDF15* is known to be TP53-controlled, DNA damage-responsive, pro-cell death gene induced by variety of stressors in human cells [31,32]. Importantly, *GDF15* is among the four genes reported to be needed to accurately perform biodosimetry in human peripheral blood cells [33]; therefore, the level of expression of this gene may reflect the ultimate cell fate after IR exposures. Noteworthy, *GDF15* tops the list of the most upregulated genes induced as an essential part of an early radioresponse to HDIR in all four hESCs lines studied, and *BTG2* consistently scoring the second. *BTG2* halts cell proliferation; and it is transiently triggered by oxidative stress involving both TP53 and NF-κB pathways, stimulating antioxidant defenses and repair of DNA double-strand breaks (DSBs) [34]. We confirmed the validity of our DNA microarray platform with quantitative real-time PCR performed on a partial dataset of RNA species, including *CDKN1A*, in a previous study [11].

Our analysis of the patterns of repressed genes across distinct HDIR-exposed hESC lines shows that these are strikingly different from the patterns observed for overexpressed genes (Figure 2B). To illustrate this, none of the repressed genes is shared in common by all four hESC lines we studied; and only five genes were downregulated in three of these lines as an early response to HDIR. Therefore, virtually all repressed genes turn out to be hESC line-specific; however, about 50 genes are induced in all four—H1, H7, H9 and H14—hESC lines, and many more genes are shared in common by a combinations of three lines out of four examined (Figure 2). Unfortunately, previous research has not focused much on how different lines of hESCs respond to IR exposures on global gene expression level. However, the difference in behavior of distinct human cell lines in response to IR has been reported many times before, including by us (for example, see [21,35]). The repression of genes in response to IR in hESCs, such as H9, has been examined in a previous study, with doses of IR exposures spanning from 0.4 to 4 Gy [19]. The cell-type specificity of gene expression alterations may depend on differential genomic/epigenomic landscapes in the distinct human cell lines, potentially translating into differences in global gene expression as a result of IR exposures. Our findings imply that the molecular mechanisms of gene expression changes elicited by HDIR might be distinct for different modes of transcript regulation justifying further research in this direction.

**Figure 3:**
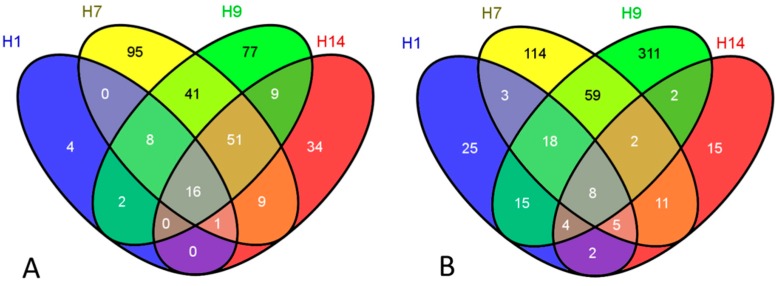
Venn diagrams of differentially expressed genes following HDIR exposures (16 h): (**A**) induced genes and (**B**) repressed genes.

We identified 31 genes in H1 hESCs; 221 genes in H7; 204 genes in H9; and 120 genes in H14 hESCs as overexpressed after 16 h post HDIR (Figure 3A). Among these genes, 16 genes appear to be shared in common by all four hESC lines; for example, *GDF15*, *BTG2*, *ACTA2*, *PHLDA3*, *CDKN1A*, *etc.* belong to this group. Notably, the level of expression of these genes 16 h post HDIR is much lower compared to an early response to HDIR (2 h). If H1 hESC line is not taking into consideration, another subset of 51 genes common to H7, H9, and H14 hESC lines is identified (Figure 3A). Therefore, the gene expression profile of H1 hESC line is somewhat distinct under these conditions. In addition, our microarray analysis showed that 80 genes in H1 hESCs; 220 genes in H7 hESCs; 415 genes in H9 hESCs; and 49 genes in H14 line of hESC were repressed 16 h post HDIR; and only eight genes were found in common across all these lines (Figure 3B).

To elucidate the biological processes/pathways that were among the most affected by gene expression alterations following IR exposures of hESCs, we conducted Gene Ontology analysis for both overexpressed and repressed genes separately. This study was performed on a whole set of statistically significant differentially expressed genes shown in Figure 1, Figure 2 and Figure 3. The biological processes primarily affected by both LDIR and HDIR in H1, H7, H9 and H14 hESC lines are indicated in Supplementary Tables S1–S4. These ranged from general metabolic processes, such as cholesterol biosynthesis and alternative splicing, to cell redox homeostasis, cell cytoskeleton, and cell adhesion. Many of the biological processes/themes/pathways were unique to a specific condition, or a cell line, but some were found to be induced/repressed across multiple cell lines. Therefore, the results of our analysis imply that there are common biological themes between experimental groups. Notably, p53 signaling pathway, and cell cycle arrest are induced as in integral part of an early (2 h) radioresponse to HDIR in H1-H7-H9-H14; positive regulation of apoptosis is overrepresented in H1-H9-H14 lines; cellular response to stress in H1-H7-H9; and activation of caspase activity in H7-H9-H14 hESC lines.

The late (16 h) response to HDIR involves the induction of a distinct subset of processes/pathways in hESCs compared to an early one. Importantly, none of those are in common across all four lines examined; however, p53 signaling pathway, positive regulation of apoptosis, and negative regulation of cell proliferation are overrepresented in H7-H9-H14 lines of hESC. Interestingly, we found consistent upregulation of metallothionein superfamily at 16 h post-HDIR in H1-H7-H9 hESC lines, which is in concert with our previous work with HDIR-exposed human fibroblasts and hESCs [12,20]. This finding illustrates the principle that the upregulation of metallothioneins could be the integral part of the pro-survival strategy in different types of human cells, including hESCs. In marked agreement with the current view that for DNA repair and signaling to occur, the chromatin of HDIR-exposed cells needs to be relaxed, at least locally, we found that the DNA packaging process was repressed in common in H7-H9-H14 lines of hESC at 16 h post HDIR.

It is important to indicate that the genes implicated in DNA damage response and/or DNA repair were significantly overrepresented across all four hESC lines after HDIR based on consensus sets of statistically significant differentially expressed genes (Table 1), and in some instances, following LDIR (Supplementary Tables S1–S4). The most recent statistical analysis reinforced the earlier viewpoint that the transcription in mammalian cells could be the most important step in the complicated maze of gene expression regulation, determining the protein abundance in those cells [36]. Therefore, our DNA microarray data on changes in global gene expression across distinct hESC lines can potentially underscore not only the biological processes affected at the transcript level, but also provide clues to the importance of alterations in levels of specific gene products in radioresponses of these cells.

**Table 1:** Gene Ontology analysis of affected biological processes/pathways/themes in hESCs after HDIR based on consensus sets of genes found in all lines of hESC examined.

Exposures	Over-Represented Categories (Upregulation)	EASE Score
1 Gy, 2 h	P53 signaling pathway	7.9 × 10^−12^
	Response to DNA damage stimulus	1.5 × 10^−7^
	Positive regulation of apoptosis	5.6 × 10^−7^
	Cellular response to stress	6.7 × 10^−6^
	Negative regulation of cell proliferation	1.1 × 10^−4^
	Cell cycle arrest	2.8 × 10^−4^
	Response to radiation	3.7 × 10^−4^
	Regulation of protein kinase activity	4.1 × 10^−3^
	Regulation of transferase activity	5.6 × 10^−3^
1 Gy, 16 h	Positive regulation of anti-apoptosis	4.3 × 10^−4^
	Response to DNA damage stimulus	6.0 × 10^−3^
	Cellular response to stress	0.019
**Exposures**	**Over-Represented Categories (Downregulation)**	**EASE Score**
1 Gy, 16 h	Cytoplasmic vesicle	0.033

## 3. Experimental Section

### 3.1. Cell Culture

A panel of cultured hESCs (H1, H7, H9 and H14 cell lines, WiCell, Madison, WI, USA) was routinely grown in mTeSR-1 medium (Stemcell Technologies, Vancouver, BC, Canada) on a BD Matrigel hESC-qualified matrix (BD Biosciences, San Jose, CA, USA) in a humidified 5% CO_2_ incubator at 37 °C, as in [20,21,37]. Cells were subcultured every 5–7 days using dispase (Stemcell Technologies). Cell cultures were maintained and propagated according to the supplier’s protocol.

### 3.2. Cell Culture Treatments

The irradiation of cell cultures was performed using an Eldorado 8 ^60^Co teletherapy unit (MDS Nordion, Ottawa, ON, Canada; formerly Atomic Energy of Canada, Ltd.). The cells were exposed to 0.05, and 1 Gy of γ-radiation, and then allowed to recover in a CO_2_ incubator for either 2 or 16 h, as in [11]. In parallel, the control cell cultures were treated with sham-radiation (Eldorado 8 ^60^Co teletherapy unit, MDS Nordion). At the indicated time points post-IR, the cell cultures were lysed with Trizol (Life Technologies, Grand Island, NY, USA); and the samples were processed as per manufacturer’s protocol for downstream analysis. All experiments were performed in quadruplicates.

### 3.3. RNA Sample Preparation, Probe Labeling and DNA Microarray Procedure

Total RNA was extracted using Trizol (Invitrogen), and then purified with RNeasy kit (Qiagen, Valencia, CA, USA) and TURBO DNA-free kit (Ambion, Austin, TX, USA), as described in [12]. The amount and quality of RNA preparations were evaluated on the Agilent 2100 Bioanalyzer with RNA 6000 Nano Reagents and Supplies (Agilent, Santa Clara, CA, USA). Agilent RNA Spike-In Mix was added to the RNA samples prior to the labeling reactions following the RNA Spike-In Kit protocol. Subsequently, cRNA targets were synthesized from 0.1 μg of total RNA in each reaction and fluorescently labeled with either Cy5-CTP or Cy3-CTP (PerkinElmer, Waltham, MA, USA) in separate labeling reactions using the Agilent Low Input Quick-Amp Labeling kit. The dual-labeled cRNA targets corresponding both to experimental and control samples were combined and hybridized to 4 × 44 k Agilent Human Whole Genome oligo microarrays using Agilent SureHyb hybridization chambers. Protocols for microarray hybridization and washing were as suggested by manufacturer. Hybridized DNA microarrays were scanned on an Agilent SureScan DNA microarray scanner, and TIFF images were subsequently processed by Feature Extraction 12.0 software (Agilent). All samples had four independent biological replicates, and each replicate was run on a separate array. The median normalization approach was applied to the raw data. Each data point was represented by four independent biological replicates, and each replicate was run on separate DNA microarray slide as in [12,20,35].

### 3.4. Data Statistical Analysis

Analyses were performed using BRB-Array Tools 4.4.0 Beta_1 developed by Dr. Richard Simon and BRB-Array Tools Development Team (Biometric Research Branch, National Cancer Institute, NIH, Bethesda, MD, USA). Differentially expressed genes were identified using ANOVA test; and the *p*-value was set to be less than 0.001 for genes to be considered differentially expressed. Threshold false discovery rate (FDR) for differentially expressed genes was set at 0.1. The Gene Ontology analysis was performed using the DAVID software (National Institute of Allergy and Infectious Diseases, NIH) [38]. The biological themes/processes with EASE scores less than 0.05 were considered to be statistically significant [39,40].

## 4. Conclusions

By using systems biology approaches, we examined, for the first time, the impact of low, clinical diagnostic relevant (LDIR), and HDIR exposures in modulating the transcriptional responses of different lines of cultured hESCs. We report here that the whole genome gene chip technique is capable of detecting statistically significant changes in gene expression in hESCs even after doses of IR exposures as low as five rad (LDIR). “Early” (2 h) gene expression signatures of hESCs after LDIR involves the consistent induction of only one gene (*CDKN1A*) across all lines of hESC examined; the subset of other LDIR-responsive genes are found to be cell line-specific, with the level of induction not more than 2.5-fold change. The sets of down-regulated LDIR genes across hESCs are more abundant and diverse, compared to mostly p53-regulated induced genes. Except H7 hESC line, no genes are found to be statistically significantly differentially expressed after 16 h post-LDIR in studied hESCs pointing to a transient gene expression changes in these hESCs. “Early” (2 h) gene expression signatures of hESCs after 1 Gy (HDR) involves the consistent induction of 50 genes across all lines of hESC examined (Supplementary Table S5), with the highest level of induction up to 24-fold seen for *GDF15*; the big subset of these 50 genes are p53-regulated. Interestingly, almost all repressed genes are found to be cell line-specific after HDR. The common repertoire of induced genes after 16 h post-HDR constituting the “late” response is more limited compared to “early” response (only 16 genes shared by all lines of hESC studied, with the highest level of induction about threefold over control). The vast majority of genes are cell-type specific. Gene expression signatures of hESC exposed to IR appear to be highly dose-, time-, and cell line-dependent. Moreover, the biological processes, pathways and themes implicated in a radioresponse of human embryonic stem cells were identified, among them p53 signaling pathway and cell cycle arrest were common to all hESC lines after HDIR; these data could potentially serve as a guide to developing novel predictive biomarkers of the deleterious effects of IR upon clinically diagnostic relevant human exposures and to improve the models for IR-related risk assessment for personalized therapy.

## Supplementary Materials

Supplementary materials can be found at http://www.mdpi.com/1422-0067/16/07/14737/s1.
