# Supplementary material for: Comparative Analysis of Whole-Genome Gene Expression Changes in Cultured Human Embryonic Stem Cells in Response to Low, Clinical Diagnostic Relevant, and High Doses of Ionizing Radiation Exposure
Source: Int J Mol Sci. 2015 Jun 30;16(7):14737–48. doi: 10.3390/ijms160714737 (PMC4519869; doi:10.3390/ijms160714737)
Supplement: Supplementary file 1 [file ijms-16-14737-s001.pdf]

# Supplementary Information

**Table S1.** Gene Ontology analysis of affected biological processes/pathways/themes in H1 hESCs based on sets of statistically significant differentially expressed genes.

| Exposures | Overrepresented Categories (Upregulation) | EASE Score |
|---|---|---|
| 5 cGy, 2 h | Negative regulation of cell differentiation | 0.0049 |
| | Lipid biosynthetic process | 0.015 |
| | Negative regulation of cell proliferation | 0.02 |
| | Transcription factor binding | 0.037 |
| | Regulation of apoptosis | 0.038 |
| | Positive regulation of anti-apoptosis | 0.048 |
| 1 Gy, 2 h | P53 signaling pathway | $4.2 \times 10^{-10}$ |
| | Positive regulation of apoptosis | $7.5 \times 10^{-8}$ |
| | Response to DNA damage stimulus | $1.5 \times 10^{-6}$ |
| | Cellular response to stress | $9.6 \times 10^{-6}$ |
| | Negative regulation of cell proliferation | $9.8 \times 10^{-6}$ |
| | Cell cycle arrest | $7.0 \times 10^{-4}$ |
| | Negative regulation of cell differentiation | $1.5 \times 10^{-3}$ |
| | Regulation of protein kinase activity | 0.011 |
| | I-kappaB kinase/NF-kappaB cascade | 0.025 |
| 1 Gy, 16 h | Metallothionein superfamily | $9.9 \times 10^{-18}$ |
| | Induction of apoptosis | $8.8 \times 10^{-5}$ |
| | DNA damage response | $2.6 \times 10^{-4}$ |
| | Positive regulation of anti-apoptosis | 0.001 |
| | Positive regulation of cell death | 0.005 |
| | Cellular response to stress | 0.012 |
| **Exposures** | **Overrepresented Categories (Downregulation)** | **EASE Score** |
| 1 Gy, 2 h | Alternative splicing | 0.016 |
| | Chromatin organization | 0.020 |
| 1 Gy, 16 h | Chromatin assembly | $2.0 \times 10^{-6}$ |
| | Cholesterol biosynthesis | $5.1 \times 10^{-5}$ |
| | Macromolecular complex assembly | $9.3 \times 10^{-4}$ |
| | PPAR signaling pathway | 0.007 |
| | Hemopoietic organ development | 0.033 |
| | Immune system development | 0.040 |

**Table S2.** Gene Ontology analysis of affected biological processes/pathways/themes in H7 based on sets of statistically significant differentially expressed genes.

| Exposures | Overrepresented Categories (Upregulation) | EASE Score |
|---|---|---|
| 1 Gy, 2 h | Regulation of programmed cell death | $2.8 \times 10^{-10}$ |
| | Apoptosis | $3.8 \times 10^{-10}$ |
| | P53 signaling pathway | $5.0 \times 10^{-10}$ |
| | Response to DNA damage stimulus | $1.6 \times 10^{-7}$ |
| | Cellular response to stress | $8.9 \times 10^{-6}$ |
| | Negative regulation of cell proliferation | $1.7 \times 10^{-5}$ |
| | Negative regulation of apoptosis | $2.9 \times 10^{-4}$ |
| | Negative regulation of mitotic cell cycle | $9.1 \times 10^{-4}$ |
| | Activation of caspase activity | 0.001 |
| | Cell cycle arrest | 0.002 |
| | Protein import into nucleus | 0.003 |
| | Positive regulation of hydrolase activity | 0.005 |
| | Response to radiation | 0.008 |
| | Transcription repressor activity | 0.009 |
| | Negative regulation of cell size | 0.011 |
| | Negative regulation of cell differentiation | 0.012 |
| | Intracellular signaling cascade | 0.013 |
| | Positive regulation of gene expression | 0.032 |
| | DNA binding | 0.034 |
| | DNA repair | 0.039 |
| 1 Gy, 16 h | P53 signaling pathway | $3.4 \times 10^{-8}$ |
| | Metallothionein superfamily | $1.5 \times 10^{-7}$ |
| | Positive regulation of apoptosis | $6.1 \times 10^{-7}$ |
| | Negative regulation of cell proliferation | $3.8 \times 10^{-6}$ |
| | Response to steroid hormone stimulus | $6.6 \times 10^{-6}$ |
| | Negative regulation of cell differentiation | $2.2 \times 10^{-5}$ |
| | Cell motion | $3.8 \times 10^{-5}$ |
| | Response to glucocorticoid stimulus | $6.1 \times 10^{-5}$ |
| | Enzyme linked receptor protein signaling pathway | $1.3 \times 10^{-4}$ |
| | Actin cytoskeleton | $2.2 \times 10^{-4}$ |
| | Positive regulation of cell proliferation | $2.8 \times 10^{-4}$ |
| | Positive regulation of cell differentiation | $7.3 \times 10^{-4}$ |
| | TGF-beta signaling pathway | 0.001 |
| | Blood vessel development | 0.001 |
| | Muscle organ development | 0.002 |
| | Response to hypoxia | 0.002 |
| | Cell migration | 0.003 |
| | Cellular response to stress | 0.006 |
| | Neurotrophin signaling pathway | 0.009 |
| | Negative regulation of apoptosis | 0.02 |
| | Response to oxidative stress | 0.02 |
| | Response to DNA damage | 0.02 |
| | Cytoskeleton organization | 0.02 |
| | Cell adhesion | 0.03 |

**Table S2.** *Cont.*

| Exposures | Overrepresented Categories (Downregulation) | EASE Score |
|---|---|---|
| 5 cGy, 2 h | M phase (of cell cycle) | $3.9 \times 10^{-5}$ |
| | DNA repair | $4.6 \times 10^{-4}$ |
| | Mitosis | $7.9 \times 10^{-4}$ |
| | Organelle fission | 0.001 |
| | Response to radiation | 0.002 |
| | Response to DNA damage stimulus | 0.002 |
| | DNA recombination | 0.014 |
| | Regulation of translation | 0.038 |
| | Mismatch repair | 0.044 |
| 1 Gy, 2 h | Chromosome organization | $2.2 \times 10^{-4}$ |
| | Vasculogenesis | $6.1 \times 10^{-4}$ |
| | Skeletal system development | $9.5 \times 10^{-4}$ |
| | Regulation of cell cycle | 0.001 |
| | Negative regulation of macromolecule metabolic process | 0.002 |
| | Hemopoietic organ development | 0.003 |
| | Regulation of Notch signaling pathway | 0.005 |
| | Positive regulation of cell proliferation | 0.006 |
| | Positive regulation of apoptosis | 0.008 |
| | Positive regulation of transcription | 0.009 |
| | Negative regulation of cell communication | 0.01 |
| | Protein catabolic process | 0.02 |
| | Negative regulation of gene expression | 0.02 |
| | Alternative splicing | 0.04 |
| 1 Gy, 16 h | DNA packaging | $1.7 \times 10^{-12}$ |
| | Chromosome organization | $1.8 \times 10^{-9}$ |
| | Histone H2B | $1.1 \times 10^{-8}$ |
| | Histone H2A | 0.001 |
| | Histone H1/H5 | 0.01 |
| | Regulation of neurological system process | 0.02 |
| | Negative regulation of apoptosis | 0.04 |
| | Positive regulation of hydrolase activity | 0.04 |

**Table S3.** Gene Ontology analysis of affected biological processes/pathways/themes in H9 based on sets of statistically significant differentially expressed genes.

| Exposures | Overrepresented Categories (Upregulation) | EASE Score |
|---|---|---|
| 5 cGy, 2 h | Regulation of apoptosis | 0.0012 |
| | Response to DNA damage stimulus | 0.0015 |
| | Cellular response to stress | 0.0051 |
| | Negative regulation of cell proliferation | 0.023 |
| | P53 signaling pathway | 0.05 |
| 1 Gy, 2 h | Positive regulation of apoptosis | $2.4 \times 10^{-10}$ |
| | P53 signaling pathway | $1.2 \times 10^{-9}$ |
| | DNA damage response | $1.3 \times 10^{-8}$ |
| | Cellular response to stress | $6.3 \times 10^{-7}$ |
| | Activation of caspase activity | $4.1 \times 10^{-4}$ |
| | Cell cycle arrest | $5.5 \times 10^{-4}$ |
| | DNA repair | $2.5 \times 10^{-3}$ |
| | DNA damage checkpoint | $3.7 \times 10^{-3}$ |
| | Positive regulation of macromolecule biosynthesis | $3.3 \times 10^{-3}$ |
| 1 Gy, 16 h | Metallothionein superfamily | $8.3 \times 10^{-14}$ |
| | Positive regulation of apoptosis | $1.0 \times 10^{-5}$ |
| | P53 signaling pathway | $6.4 \times 10^{-5}$ |
| | TGF-beta signaling pathway | $6.6 \times 10^{-5}$ |
| | Extracellular matrix | $1.2 \times 10^{-4}$ |
| | Negative regulation of cell proliferation | $3.0 \times 10^{-4}$ |
| | Response to oxidative stress | $6.5 \times 10^{-4}$ |
| | Cytoskeleton organization | 0.0049 |
| **Exposures** | **Overrepresented Categories (Downregulation)** | **EASE Score** |
| 5 cGy, 2 h | Nucleosome | $1.7 \times 10^{-5}$ |
| | Histone H2A | $2.2 \times 10^{-5}$ |
| | Nucleosome assembly | $1.1 \times 10^{-4}$ |
| | DNA packaging | $3.8 \times 10^{-4}$ |
| | Cytoplasmic vesicle | 0.021 |
| 1 Gy, 2 h | Negative regulation of cell communication | $5.4 \times 10^{-7}$ |
| | Skeletal system development | $4.4 \times 10^{-4}$ |
| | Regulation of cell proliferation | $4.8 \times 10^{-3}$ |
| | Negative regulation of macromolecule biosynthesis | $4.8 \times 10^{-3}$ |
| | Regulation of transcription | $7.5 \times 10^{-3}$ |
| | Positive regulation of cell differentiation | 0.011 |
| | Protein kinase cascade | 0.04 |
| 1 Gy, 16 h | mRNA processing | $5.7 \times 10^{-4}$ |
| | Sterol biosynthetic process | $8.4 \times 10^{-4}$ |
| | RNA splicing | 0.0012 |
| | Cholesterol biosynthesis | 0.0068 |
| | RNA export from nucleus | 0.011 |
| | M phase of mitotic cell cycle | 0.023 |
| | DNA packaging | 0.04 |

**Table S4.** Gene Ontology analysis of affected biological processes/pathways/themes in H14 based on sets of statistically significant differentially expressed genes.

| Exposures | Overrepresented Categories (Upregulation) | EASE Score |
|---|---|---|
| 5 cGy, 2 h | Response to DNA damage stimulus | 0.00033 |
| | P53 signaling pathway | 0.0026 |
| | Cell cycle arrest | 0.0042 |
| | Wnt signaling pathway | 0.012 |
| | Response to radiation | 0.015 |
| | DNA repair | 0.029 |
| | Negative regulation of apoptosis | 0.044 |
| 1 Gy, 2 h | P53 signaling pathway | $1.0 \times 10^{-12}$ |
| | Positive regulation of apoptosis | $2.0 \times 10^{-9}$ |
| | DNA damage response | $5.3 \times 10^{-7}$ |
| | Release of cytochrome c from mitochondria | $4.6 \times 10^{-5}$ |
| | Activation of caspase activity | $8.9 \times 10^{-5}$ |
| | Cell cycle arrest | $2.0 \times 10^{-4}$ |
| | Cell migration | $1.2 \times 10^{-3}$ |
| | MAPK signaling pathway | 0.0073 |
| 1 Gy, 16 h | Positive regulation of apoptosis | $5.2 \times 10^{-10}$ |
| | P53 signaling pathway | $2.3 \times 10^{-6}$ |
| | DNA damage response | $2.7 \times 10^{-5}$ |
| | Negative regulation of cell differentiation | $6.7 \times 10^{-4}$ |
| | Negative regulation of cell proliferation | $7.7 \times 10^{-4}$ |
| | Response to wounding | $9.1 \times 10^{-4}$ |
| | Inflammatory response | 0.0066 |
| **Exposures** | **Overrepresented Categories (Downregulation)** | **EASE Score** |
| 1 Gy, 2 h | Blood vessel development | 0.0018 |
| | Neural crest development | 0.034 |
| 1 Gy, 16 h | DNA packaging | $1.2 \times 10^{-17}$ |
| | Citrullination | $3.7 \times 10^{-15}$ |
| | Histone H2A | $8.1 \times 10^{-13}$ |
| | Hemopoiesis | 0.016 |

**Table S5.** Genes upregulated in all hESC lines (H1, H7, H9 and H14) after 1 Gy IR exposures (2 h post IR). $p < 0.001$.

GDF15
BTG2
PLK2
CDKN1A
PHLDA3
SESN1
BBC3
GADD45A
TP53INP1
DRAM1
PLK3

GRHL3
ZNF79
PPM1D
TNFRSF10B
KITLG
FAM212B
ASCC3
SERTAD1
FDXR
TNFRSF10A
POLH
TOB1
PDE4A
C12orf5
RGS16
TRIM22
AEN
ARC
BCL3
DDB2
IER5
FOSL1
PTP4A1
UFM1
HES1
IKBIP
PMAIP1
STK17A
VWCE
SESN2
BDNF
E2F7
TRIAP1
BRMS1L
DDIT4
ARL4A
ANKRA2
CHADL
EPHA2

Genes upregulated in all hESC lines (H1, H7, H9 and H14) after 1 Gy IR exposures (16 h post IR). $p < 0.001$.

GDF15
BBS9
BTG2
ACTA2
PHLDA3
PLK2
CDKN1A
EPS8L2
TNFRSF10C
FDXR
RPS27L
INPP5D
GNA14
SULF2
DRAM1
ITIH5

Genes downregulated in all hESC lines (H1, H7, H9 and H14) after 1 Gy IR exposures (16 h post IR). $p < 0.001$.

SEMG1
HIST1H1D
LEAP2
ATP8B3
PNMA2
USP3
SLC1A3
NPTX1
